# Necrotizing fasciitis as the initial presentation of disseminated infection with fluconazole-resistant *Cryptococcus neoformans*

**DOI:** 10.1099/jmmcr.0.003608-0

**Published:** 2014-12-01

**Authors:** Timothy E Richardson, Nathan E Lee, Matthew D Cykowski, Spencer A Chang, Suzanne Z Powell

**Affiliations:** ^1^​ Texas College of Osteopathic Medicine and The Department of Pharmacology & Neuroscience, University of North Texas Health Science Center, Fort Worth, TX 76107, USA; ^2^​ Department of Pathology and Genomic Medicine, Houston Methodist Hospital, Weill Cornell Medical College, Houston, TX 77030, USA; ^3^​ Department of Radiology, Houston Methodist Hospital, Weill Cornell Medical College, Houston, TX 77030, USA

**Keywords:** Keywords: cryptococcosis, fluconazole resistance, fungal fasciitis.

## Abstract

**Introduction:**

*Cryptococcus neoformans* is an encapsulated budding yeast that is a common cause of opportunistic infections, rarely giving rise to cellulitis, vasculitis or fasciitis. Necrotizing fasciitis caused by *C. neoformans* is a rare but serious problem in post-transplant immunosuppression.

**Case presentation:**

We report a case of cryptococcal necrotizing fasciitis in the left adductor longus of a patient on immunosuppressive therapy. The patient’s medical history was significant for orthotopic heart transplant secondary to cardiac and systemic amyloidosis (AL type) with subsequent cardiac biopsy demonstrating mild rejection (grade 1R). A thigh muscle biopsy demonstrated numerous encapsulated fungi in the fascia and no evidence of myositis. Cryptococcal antigen was subsequently identified in the patient’s serum and cerebrospinal fluid. The patient progressed to involvement of the central nervous system, left biceps femoris and skin of the left lower leg by fluconazole-resistant *C. neoformans*.

**Conclusion:**

This case illustrates a rare initial presentation of disseminated fluconazole-resistant *C. neoformans* as an isolated necrotizing fasciitis of the thigh. Necrotizing fungal fasciitis should be considered in immunosuppressed patients with clinical findings of either myositis or cellulitis of a lower extremity.

## Introduction

*Cryptococcus neoformans* is a pathogenic and encapsulated budding yeast that consists of three known serotypes and four molecular subtypes (Chayakulkeeree & Perfect, 2006). Human exposure frequently comes through the inhalation of yeast forms in specific environmental settings (e.g. soil enriched by bird droppings, tree hollows) (Chayakulkeeree & Perfect, 2006; Mitchell & Perfect, 1995). This typically results in subclinical infection except in the setting of immunosuppression, where the organism may be a significant cause of morbidity and mortality (e.g. up to a 25 % mortality rate in cryptococcal meningitis associated with human immunodeficiency virus (HIV/AIDS)) (Saag *et al.*, 2000). Up to 95 % of clinically significant cryptococcal infections have been attributed to *C. neoformans* var. *grubii* (serotype A) (Chayakulkeeree & Perfect, 2006). Predisposing conditions include immunomodulatory therapy (e.g. autoimmune disease, post-transplant), haematological malignancies, primary or acquired immunodeficiencies, and hepatitis C (Baer *et al.*, 2009; Basaran *et al.*, 2003; Crum–Cianflone, 2008). Conditions less commonly associated with disseminated cryptococcosis include diabetes, renal failure, cirrhosis and poor nutritional state (Wang & Lim, 2014), as well as pregnancy (Chayakulkeeree & Perfect, 2006). Patients with HIV/AIDS account for a significant proportion of cases (Mitchell & Perfect, 1995; Murakawa *et al.*, 1996).

Although the majority of infections with *Cryptococcus* remain confined to the lungs (Basaran *et al.*, 2003), rare cases may also demonstrate soft-tissue involvement in the form of cellulitis, vasculitis (Shrader *et al.*, 1986), myositis or fasciitis (Adachi *et al.*, 2009; Baer *et al.*, 2009; Begon *et al.*, 2009; Marcus *et al.*, 1998). The majority of these fungal infections in deep soft tissues have been described in case reports, as these are uncommon (Crum–Cianflone, 2008). A significant clinical issue in such cases is that the clinical symptoms and signs in these patients mimic other disease processes. The signs and symptoms of necrotizing fasciitis are non-specific and include fever, erythema overlying the infection site, discharge and eventually sepsis (Buchanan *et al.*, 2013). Cutaneous lesions of *Cryptococcus*, occurring in approximately 10 % of patients with disseminated cryptococcosis (Shrader *et al.*, 1986), as well as in primary cutaneous cryptococcosis (Spiliopoulou *et al.*, 2012), are also non-specific and range from abscess to cellulitis to umbilicated nodules (Baer *et al.*, 2009). As such, cases with superficial soft-tissue and/or skin involvement can closely mimic acute bacterial cellulitis (Kimura *et al.*, 2001). Likewise, a soft-tissue fasciitis in the setting of cryptococcosis may suggest group A *Streptococcus*, methicillin-resistant *Staphylococcus aureus*, *Clostridium* spp. or a combination of Gram-negative and anaerobic bacterial organisms (Baer *et al.*, 2009; Basaran *et al.*, 2003; Wang & Lim, 2014). Treatment for a presumptive bacterial infection in these settings may lead to broad-spectrum antibiotic therapy that does not cover *Cryptococcus* spp. (Baer *et al.*, 2009; Kimura *et al.*, 2001; Spiliopoulou *et al.*, 2012). Moreover, these cases are critical to recognize early, as up to 60 % of patients presenting with soft-tissue infection will succumb to disseminated cryptococcosis (Baer *et al.*, 2009).

Here, we report a case of cryptococcal necrotizing fasciitis of the left posteromedial thigh. The patient was 6 months status post-orthotopic heart transplant for AL-type amyloidosis and was on immunosuppressive therapy secondary to mild transplant rejection. The *Cryptococcus* organism isolated proved to be fluconazole resistant. This highlights a rare clinical presentation of disseminated cryptococcosis as well as the issue of fluconazole resistance in this setting.

## Case report

**Fig. 1.**
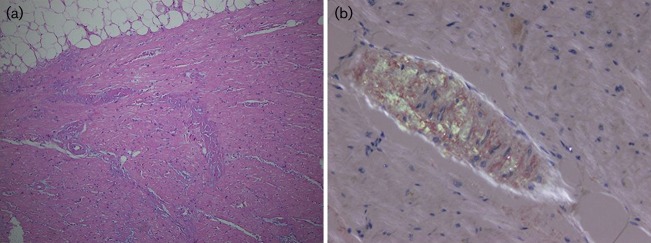
Amyloid deposition in the explanted heart. (a) Left ventricle of the explanted heart demonstrating amorphous eosinophilic material within vessel walls and between adjacent cardiac myocytes. (b) Polarization of a Congo red-stained slide demonstrating the classic apple green birefringence of amyloid.

The patient was a 51-year-old male with a history of systemic amyloidosis and non-ischaemic cardiomyopathy. Other significant past medical history included chronic kidney disease, lower extremity venous thrombosis, migraine and tension headache, and a pacemaker implant for sinus node dysfunction. A cardiac biopsy 10 months prior to the case described here revealed diffuse myocardial amyloid deposition and diffuse cardiomyocyte atrophy. Further subtyping (performed at the Mayo Clinic, Rochester, MN, USA) demonstrated light-chain (AL) amyloid. A bone-marrow biopsy with flow cytometry analysis demonstrated plasma cells (6–7 % and 2 % of total cell population on biopsy and flow cytometry, respectively) with λ light-chain restriction. Orthotopic heart transplantation was performed 4 months after the cardiac biopsy. The explanted heart demonstrated biventricular hypertrophy, thickening of the interventricular septum and diffuse amyloid deposition (Fig. 1).

One week after cardiac transplantation, an endomyocardial biopsy demonstrated features of mild rejection (grade 1R) with 5 % of capillaries staining for C4d. Approximately 4 weeks later, the patient’s biopsy continued to demonstrate mild rejection (grade 1R) and demonstrated focal myocardial necrosis with 25 % of capillaries staining positive for C4d. A biopsy performed 3 months after transplant demonstrated no evidence of acute cellular rejection (grade 0) and negative C4d staining. During this period, the patient’s immunosuppressive regimen included dexamethasone, bortezomib (for systemic amyloidosis), tacrolimus and mycophenolic acid.

**Fig. 2.**
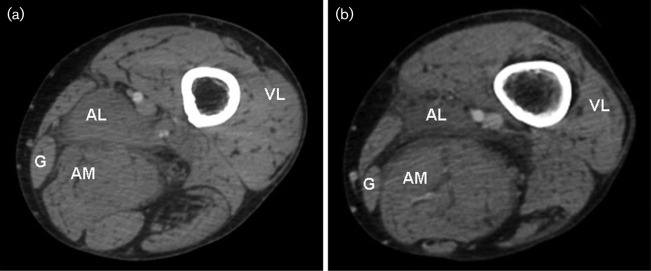
Radiological findings. (a, b) Axial CT sections through the left thigh following intravenous contrast at initial presentation with thigh pain. The posterior compartment, adductor longus (AL) and magnus (AM), gracilis (G) and vastus lateralis (VL) are visualized, as are the femoral artery and vein (in the adductor canal, medial and superficial to AL). CT examination noted increased oedema and heterogeneous density within AL and adjacent inflammatory stranding (b).

Six months after cardiac transplant, the patient presented to the hospital with fever up to 101 °F, back pain and severe left lower extremity pain. The infectious disease service was consulted, the immunosuppressive agents were decreased and empiric therapy with vancomycin was begun. Subsequent to this, laboratory results demonstrated leukocytosis (white blood cell count of 14.7×10^3^ μl^−1^) and an anti-streptococcal antibody level came back positive. Computed tomography (CT) studies with contrast demonstrated oedema and a heterogeneous density in the left adductor longus muscle with concomitant inflammatory stranding noted along the adductor longus and sciatic nerve (Fig. 2). Radiological interpretation was compatible with myositis and/or cellulitis.

**Fig. 3.**
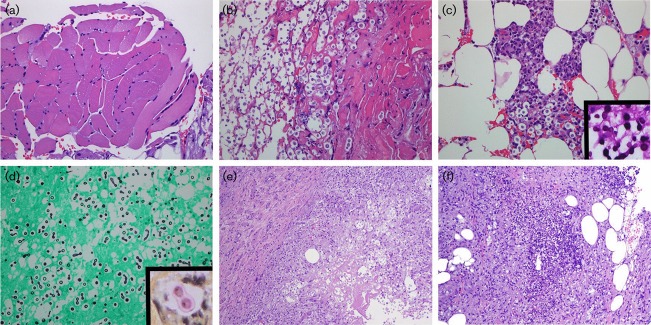
Histological findings at initial and repeat presentations. (a) Adductor longus muscle biopsied at initial presentation demonstrating mild fibre size variation and no evidence of myositis. (b–d) Biopsy of posteromedial thigh fascia demonstrating numerous fungal organisms (b) and mixed inflammatory infiltrate (c). Special stains with periodic acid–Schiff (c, inset), Grocott’s methenamine silver (d) and mucicarmine (d, inset) demonstrated encapsulated, round 5–15 µm yeasts with narrow-based budding. (e) Biopsy of the posterior thigh at repeat presentation demonstrating necrosis, granulomatous inflammation and conspicuous *Cryptococcus* organisms. (f) Necrotizing acute inflammation and fat necrosis were identified in sections of skin and subcutaneous tissue from the left leg. Grocott’s methenamine silver stain again demonstrated *Cryptococcus* organisms (not shown).

The patient’s symptoms persisted despite vancomycin therapy for presumptive bacterial cellulitis and a left lower extremity rash developed. Due to the uncertain aetiology of the radiological findings, a portion of the adductor longus muscle and surrounding upper thigh soft tissue were biopsied. The muscle showed changes of mild neurogenic atrophy (angulated myofibres, nuclear clumping, mild variation in fibre size) and focal, chronic endomysial inflammation. There was neither myositis nor evidence of amyloid deposition. The left thigh fascia showed large groups of encapsulated, narrow-based budding yeast (5–15 µm), which stained positive with mucicarmine, periodic acid–Schiff and Grocott’s methenamine silver stains, consistent with *Cryptococcus* spp. (Fig. 3).

Blood cultures revealed the presence of cryptococcal antigen, and the patient was switched to oral fluconazole and intravenous amphotericin B. After the administration of antifungal agents, the patient remained febrile and complained of persistent extremity pain. His clinical condition worsened and the patient developed hypotension with accompanying headaches. The neurology service was consulted and a lumbar puncture was performed revealing cryptococcal antigen (1 : 8 titre) in the cerebrospinal fluid (CSF), consistent with cryptococcal meningitis. The patient was continued on aggressive antifungal therapy and during this time improved significantly, eventually receiving physical therapy and rehabilitation services. Once the patient was haemodynamically stable and afebrile, he was discharged on oral antifungal therapy with micafungin.

The patient re-presented to the hospital after discharge with continued left lower extremity pain, swelling and erythema, fever and back pain. Examination revealed cellulitic changes in the left lower extremity in the region of the calf. The patient was admitted and started on voriconazole. A biopsy of the vastus lateralis and anterior and posterior thigh fascia was negative for organisms. However, necrotizing acute inflammation and cryptococcal organisms were seen in biopsies of the soft tissue of the posterior thigh and deep dermis/subcutaneous tissue of the left lower leg (Fig. 3). During this second hospitalization, the patient developed signs and symptoms of residual cryptococcal meningitis.

Aerobic, acid-fast bacilli and fungal cultures were grown from the CSF. A yeast colony suspicious for *C. neoformans* was recovered from an inhibitory mould agar slant after 4 days of incubation at 30 °C; a wet mount revealed large, round blastoconidia. The organism was identified by the API 20C system (bioMérieux) after identification failure on the matrix-assisted laser desorption-ionization time-of-flight mass spectrometry.

Sensitivity testing was performed using a colorimetric microdilution susceptibility test (Sensititre YeastOne; Thermo Scientific). The plate was incubated at 35 °C in a non-CO_2_ incubator for 72 h. The isolate was susceptible to amphotericin B (MIC 1 µg ml^−1^), itraconazole (MIC 0.12 µg ml^−1^) and voriconazole (MIC 0.06 µg ml^−1^) but resistant to fluconazole (MIC 16 µg ml^−1^). The patient was subsequently switched to intravenous amphotericin B.

The patient was admitted to the hospital again, 5 months after the initial biopsy diagnosis of cryptococcal fasciitis, for right lower extremity tenderness. Incision and drainage was performed and pathological examination disclosed an organizing haematoma. There were no fungal organisms present and wound cultures were negative.

## Discussion

The necrotizing cryptococcal fasciitis identified in the patient discussed here was unique in several respects. One unusual feature is that the patient presented initially with unilateral lower extremity pain, instead of the more common presentation with bilateral lower extremity or multiple site involvement. An additional unique feature was that the infection was limited to the fascia and soft tissue without evidence of myositis or vasculitis. On biopsy, the muscle demonstrated only mild neurogenic atrophy, although the adjacent posteromedial thigh fascia contained numerous cryptococcal organisms. Given the radiological differential of myositis at the patient’s initial presentation, this illustrates the importance of obtaining adjacent fascia/soft tissue in immunosuppressed patients with findings suggestive of soft-tissue infection. As described above, this initial presentation in the fascia of the posteromedial left thigh was followed by subsequent identification in cerebrospinal fluid and elsewhere in the ipsilateral extremity (skin and subcutaneous tissue of the leg).

The mechanism by which our patient acquired cryptococcosis is not currently known. The portal of entry of *Cryptococcus* is typically via the respiratory tract, as discussed above, although this patient had no identified pulmonary infection. Additional mechanisms include entry of the organism via the gastrointestinal tract, transplanted tissue or direct inoculation, or by reactivation of dormant yeasts in the reticuloendothelial or lymphoid systems (Chayakulkeeree & Perfect, 2006). Soft-tissue fasciitis in cryptococcosis may follow haematogenous dissemination of the organism or trauma (Jain *et al.*, 2006). Rarely, cryptococcal fasciitis has been reported at injection sites within an extremity (Huang *et al.*, 2007).

Central nervous system (CNS) involvement was a significant complication of the case described here. The patient experienced significant symptoms referable to CNS involvement developing shortly after presentation with fasciitis. CNS involvement reflects the unique neurotropism of *Cryptococcus* (Tseng *et al.*, 2012) with about 1 million cases of cryptococcal meningitis occurring worldwide every year (Perfect, 2012). The organism is thought to access the CNS via the bloodstream, although the mechanisms underlying its ability to cross the blood–brain barrier are not well understood. Postulated mechanisms include transendothelial migration of organisms, delivery of organisms into the CNS via macrophages and paracellular migration (between endothelial cells and across the capillary basement membrane) (Tseng *et al.*, 2012). Once the organism enters the CNS, a common presenting symptom is altered mental status (Baer *et al.*, 2009) and the radiological/pathological form of involvement may range from acute meningitis to meningoencephalitis to mass lesions termed ‘cryptococcomas’ (Chayakulkeeree & Perfect, 2006; Saag *et al.*, 2000).

A pertinent diagnostic issue illustrated in this case was the pathological findings of necrotizing fungal infection in the clinical setting of a possible myositis. The causes of non-infectious myositis and/or myopathic symptoms in patients are numerous and include idiopathic inflammatory myopathies (e.g. polymyositis, inclusion body myositis), collagen vascular disease, drug and toxin reactions, metabolic myopathies (e.g. mitochondrial disorders) and various dystrophies (Crum–Cianflone, 2008), as well as viral, bacterial and fungal myositis (Sewry *et al.*, 2008). Pain is a non-specific finding in this setting and is not indicative of an infectious aetiology. Both dermatomyositis and polymyositis can be associated with pain and soft-tissue swelling and may also demonstrate rapid progression with elevated creatine kinase (Sewry *et al.*, 2008). Magnetic resonance imaging findings are also non-specific in this setting, as oedema and inflammatory stranding in adjacent fat are occasionally identified in non-infectious inflammatory myopathies such as dermatomyositis (Sewry *et al.*, 2008). In the present case, however, the biopsy findings immediately excluded other diagnostic possibilities, including viral and bacterial fasciitis/myositis. The diagnostic feature on examination by haematoxylin and eosin staining was the presence of oval fungi with non-staining peripheral ‘halos’ (the polysaccharide capsule), as well as blastoconidia (buds), which are attached to the larger fungal cell by a narrow base (Shrader *et al.*, 1986). The polysaccharide capsule of *Cryptococcus* is a virulence factor that is unique to this organism and is critical to its identification on biopsy (Fig. 3). This capsule can be visualized with mucicarcime, periodic acid–Schiff and alcian blue special stains (Chayakulkeeree & Perfect, 2006; Shrader *et al.*, 1986). There are, however, poorly encapsulated forms of the organism that remain pathogenic but that better recognized on silver-based special stains (e.g. Grocott’s methenamine silver stain) (Chayakulkeeree & Perfect, 2006).

The case illustrated here also demonstrated the difficulties encountered in managing disseminated cryptococcosis with appropriate antifungal agents. Treatment options are not uniform in these patients and depend on factors such as sites involved by infection and whether the patient is immunosuppressed (Saag *et al.*, 2000). The organism serotype, molecular subtype and even species (*C. neoformans* versus *Cryptococcus gatti*) do not currently factor into treatment planning, however (Perfect, 2012). Treatment strategies in HIV-negative patients include oral fluconazole for 6–12 months (non-CNS disease), as well as amphotericin B and flucytosine for 2 weeks, followed by oral fluconazole for several months (for CNS disease) (Chayakulkeeree & Perfect, 2006). Additional agents that have been utilized include voriconazole, itraconazole, ketoconazole and miconazole (Basaran *et al.*, 2003). For patients with CNS involvement, 2 weeks of therapy with combination amphotericin/flucytosine therapy generates a sterile CSF although cryptococcal antigen titres remain elevated (Chayakulkeeree & Perfect, 2006). A concern with amphotericin is renal toxicity, but this is mitigated through the use of lipid formulations of amphotericin (Saag *et al.*, 2000). In patients with CNS disease, an additional concern is elevated intracranial pressure, which may require non-pharmacological treatments with a combination of sequential lumbar punctures or, alternatively, placement of a lumbar drain or ventriculoperitoneal shunt (Saag *et al.*, 2000). Often, as demonstrated in our case, treatment of skin and/or soft-tissue infections includes surgical debridement in addition to antifungal therapy (Baer *et al.*, 2009). Culture of these debrided tissues, including fungal cultures, is critical to identification of uncommon organisms and to determine drug resistance (Buchanan *et al.*, 2013). Furthermore, bacterial co-infection may complicate the treatment course (Baer *et al.*, 2009), which further highlights the need to culture debrided and/or biopsied tissues. A final treatment-related issue raised by this case is the management of immunosuppressive therapy post-transplant in the setting of disseminated cryptococcosis. The treatment strategy must balance the need to reduce immunosuppression in dealing with the infection with the risks of transplant rejection.

Finally, it should be noted that fungal fasciitis/myositis may occur outside the setting of immunosuppression and may be attributable to other fungal organisms. Rare infections with *Cryptococcus* spp. have been reported in immunocompetent patients (Buchanan *et al.*, 2013). Other organisms that may be implicated include *Candida* (most commonly), *Blastomyces* and *Aspergillus* (Crum–Cianflone, 2008). Additional sources of fungal soft-tissue infection in immunocompetent patients include *Aspergillus flavus*, *Apophysomyces elegans*, *Fusarium* spp. and *Curvularia brachyspora* (Buchanan *et al.*, 2013), as well as necrotizing fasciitis attributable to zygomycetes (e.g. *Apophysomyces elegans*) (Jain *et al.*, 2006). In contrast to our patient, whose biopsy demonstrated a suppurative, necrotizing soft-tissue infection (without myonecrosis), reports of zygomycosis involving fascia have demonstrated a minimal inflammatory reaction (Jain *et al.*, 2006).

The case reported here emphasizes the importance of considering fungal sources of soft-tissue infection in immunosuppressed individuals, even when the clinical appearance mimics bacterial fasciitis and/or cellulitis. As reviewed above, a diagnosis of fungal necrotizing fasciitis in an extremity is often not considered until histopathological examination of a biopsy specimen (Shrader *et al.*, 1986), as bacterial causes are much more common (Crum–Cianflone, 2008), including *Staphylococcus*, *Streptococcus*, Gram-negative and anaerobic bacteria (Giuliano *et al.*, 1977; Kotrappa *et al.*, 1996). Nonetheless, candidal and cryptococcal fasciitis/myositis, among other sources, should be considered in any patient, even with clinical and radiological findings suggestive of a bacterial source. This may speed the diagnosis of a potentially fatal condition (e.g. disseminated cryptococcosis) and allow rapid implementation of appropriate therapy.

## Abbreviations

Abbreviations: CNS: central, nervous system
CSF: cerebrospinal fluid.
